# Supplementary figures and images for: Comprehensive computational modelling of the development of mammalian cortical connectivity underlying an architectonic type principle
Source: PLoS Comput Biol. 2018 Nov 26;14(11):e1006550. doi: 10.1371/journal.pcbi.1006550 (PMC6261046; doi:10.1371/journal.pcbi.1006550)

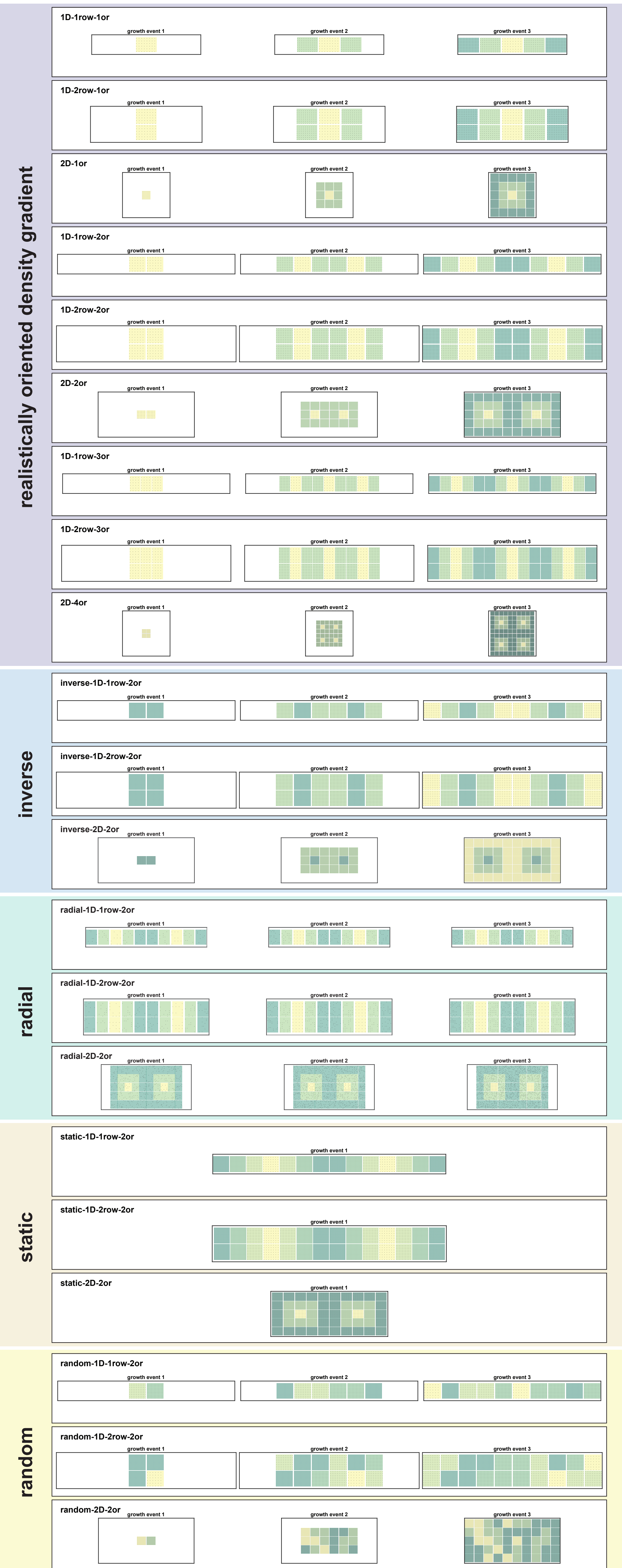

Supplement: S1 Fig — Illustration of the spatiotemporal growth trajectory for each growth layout. The successive population of the cortical sheet with neurons is shown for the first three growth events. For static growth, all neurons grow simultaneously, hence only one growth event is shown. Abbreviations and background colours as in Table 1. (PDF) [file pcbi.1006550.s001.pdf]
