# Supplementary material for: Comprehensive computational modelling of the development of mammalian cortical connectivity underlying an architectonic type principle
Source: PLoS Comput Biol. 2018 Nov 26;14(11):e1006550. doi: 10.1371/journal.pcbi.1006550 (PMC6261046; doi:10.1371/journal.pcbi.1006550)
Supplement: S2 Fig — Distribution of absent and present connections across distance (left panels) and absolute density difference (right panels) for all growth layouts. Absolute numbers of absent and present projections (bars) are depicted alongside the corresponding relative frequency of present connections (diamonds). Simulation instances were chosen to be representative of the median values shown in Fig 5. Spearman rank correlation results for each particular instance are shown on top of each plot. A.u.: arbitrary unit. Abbreviations and background colours as in Table 1. (PDF) [file pcbi.1006550.s002.pdf]

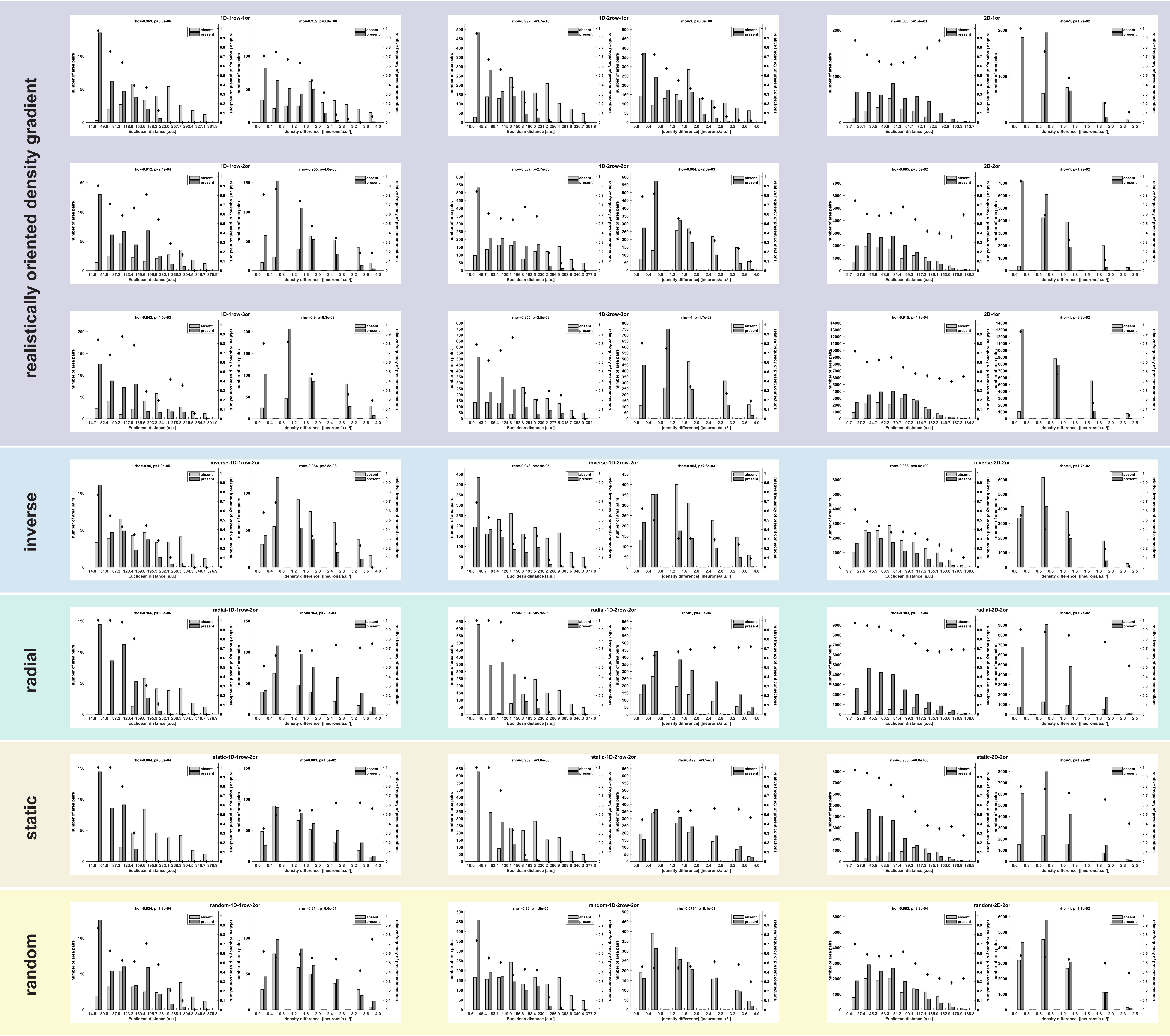

Supplementary Figure S2. Correlation relative connections frequency with distance and absolute density difference for all growth layouts. Distribution of absent and present connections across distance (left panels) and absolute density difference (right panels) for all growth layouts. Absolute numbers of absent and present projections (bars) are depicted alongside the corresponding relative frequency of present connections (diamonds). Simulation instances were chosen to be representative of the median values shown in Figure 5. Spearman rank correlation results for each particular instance are shown on top of each plot. A.u.: arbitrary unit. Abbreviations and background colours as in Table 1.
