# Supplementary material for: Comprehensive computational modelling of the development of mammalian cortical connectivity underlying an architectonic type principle
Source: PLoS Comput Biol. 2018 Nov 26;14(11):e1006550. doi: 10.1371/journal.pcbi.1006550 (PMC6261046; doi:10.1371/journal.pcbi.1006550)
Supplement: S3 Fig — Variation of area degree (number of connections) across areas’ neuron density is shown. Simulation instances were chosen to be representative of the median values shown in Fig 7. Spearman rank correlation results for each particular instance are shown on top of each plot. A.u.: arbitrary unit. Abbreviations and background colours as in Table 1. (PDF) [file pcbi.1006550.s003.pdf]

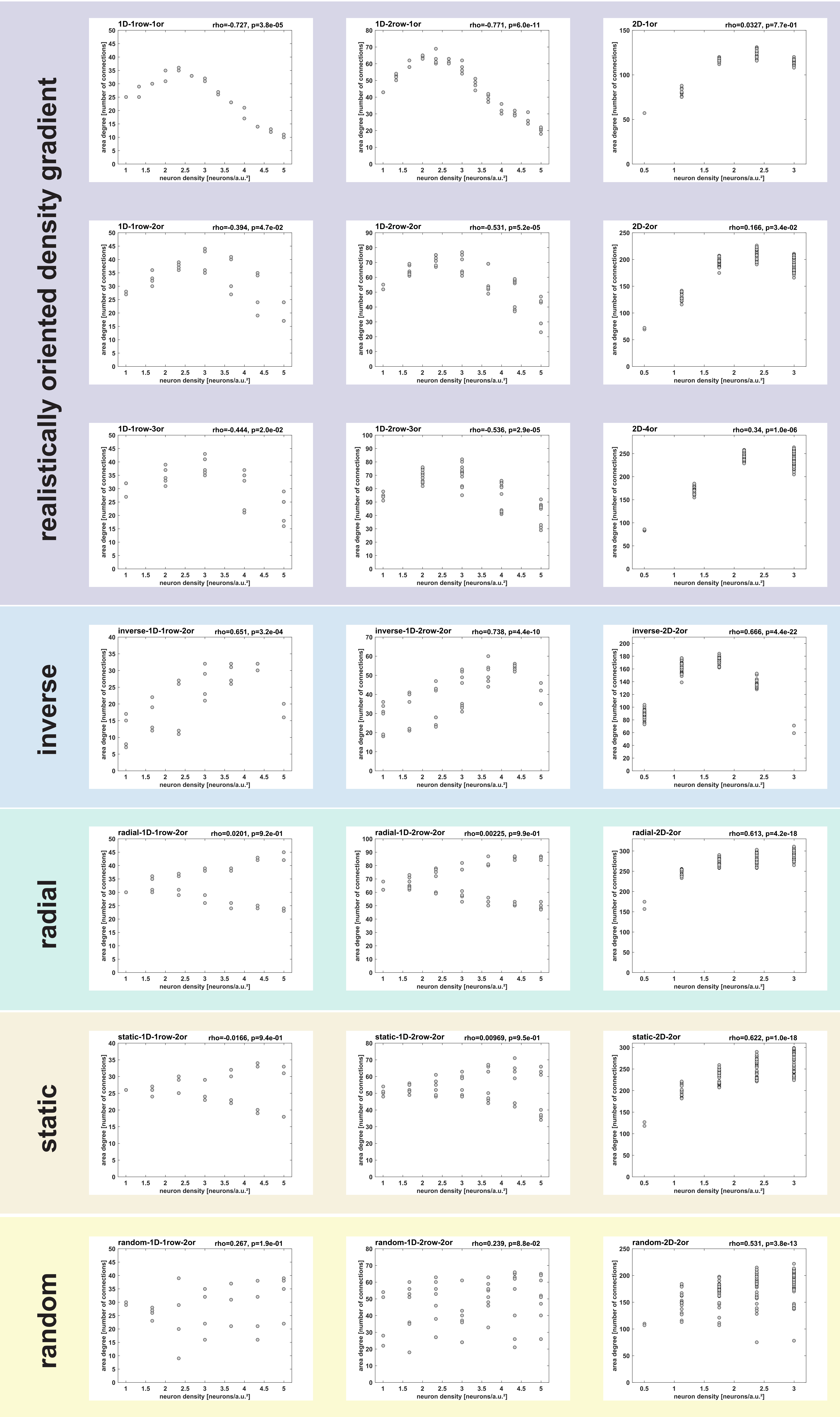

Supplementary Figure S3. Correlation area degree with neuron density for all growth layouts. Variation of area degree (number of connections) across areas' neuron density is shown. Simulation instances were chosen to be representative of the median values shown in Figure 7. Spearman rank correlation results for each particular instance are shown on top of each plot. A.u.: arbitrary unit. Abbreviations and background colours as in Table 1.

Beul et al. (2018) - Comprehensive computational modelling of the development of mammalian cortical connectivity underlying an architectonic type principle
